# Bile Acids Specifically Increase Hepatitis C Virus RNA-Replication

**DOI:** 10.1371/journal.pone.0036029

**Published:** 2012-04-25

**Authors:** Patrick Chhatwal, Dorothea Bankwitz, Juliane Gentzsch, Anne Frentzen, Philipp Schult, Volker Lohmann, Thomas Pietschmann

**Affiliations:** 1 Department of Experimental Virology, TWINCORE, Centre for Experimental and Clinical Infection Research; a joint venture between the Medical School Hannover and the Helmholtz Centre for Infection Research, Hannover, Germany; 2 Department of Infectious Diseases, Molecular Virology, University of Heidelberg, Heidelberg, Germany; Saint Louis University, United States of America

## Abstract

**Background:**

Hepatitis C virus (HCV) patients with high serum levels of bile acids (BAs) respond poorly to IFN therapy. BAs have been shown to increase RNA-replication of genotype 1 but not genotype 2a replicons. Since BAs modulate lipid metabolism including lipoprotein secretion and as HCV depends on lipids and lipoproteins during RNA-replication, virus production and cell entry, BAs may affect multiple steps of the HCV life cycle. Therefore, we analyzed the influence of BAs on individual steps of virus replication.

**Methods:**

We measured replication of subgenomic genotype (GT) 1b and 2a RNAs as well as full-length GT2a genomes in the presence of BAs using quantitative RT-PCR and luciferase assays. Cell entry was determined using HCV pseudoparticles (HCVpp). Virus assembly and release were quantified using a core-specific ELISA. Replicon chimeras were employed to characterize genotype-specific modulation of HCV by BAs. Lunet CD81/GFP-NLS-MAVS cells were used to determine infection of Con1 particles.

**Results:**

BAs increased RNA-replication of GT1b replicons up to 10-fold but had no effect on subgenomic GT2a replicons both in Huh-7 and HuH6 cells. They did not increase viral RNA translation, virus assembly and release or cell entry. Lowering replication efficiency of GT2a replicons rendered them susceptible to stimulation by BAs. Moreover, replication of full length GT1b with or without replication enhancing mutations and GT2a genomes were also stimulated by BAs.

**Conclusions:**

Bile acids specifically enhance RNA-replication. This is not limited to GT1, but also holds true for GT2a full length genomes and subgenomic replicons with low replication capacity. The increase of HCV replication by BAs may influence the efficacy of antiviral treatment in vivo and may improve replication of primary HCV genomes in cell culture.

## Introduction

Infections caused by HCV represent a serious health hazard worldwide. With ca. 160 million chronically infected patients [1] , HCV is one of the major causes of chronic liver diseases. HCV is a positive strand RNA virus with a genome of about 9.6 kb [2]. It is a highly variable virus and therefore isolates are classified into six major genotypes that differ in their nucleotide sequence by up to 35% [3]. Treatment of hepatitis C is based on a combination of pegylated interferon-α (IFN- α) and ribavirin. First viral protease inhibitors have been licensed in 2011 and substantially improve therapy response. However, since drug resistant variants are rapidly selected during monotherapy [4], these drugs complement but do not replace the previous IFN-based regimen. HCV patients that have high serum levels of BAs respond poorly to IFN therapy [5] and are more prone to develop hepatic fibrosis [6]. BAs therefore were suggested to play an important role in pathogenesis and therapy response of HCV [7], [8].

BAs are synthesized in hepatocytes using cholesterol as precursor and are then secreted from the liver via the bile duct. To increase solubility, these molecules are conjugated with glycine or taurin prior to secretion [9]. The primary BAs in humans are cholic acid (CA) and chenodeoxycholic acid (CDCA). Intestinal bacteria dehydroxylate primary BAs thus converting them to secondary BAs, such as deoxycholic acid (DCA) and lithocholic acid (LCA). A tertiary BA, ursodeoxycholic acid (UDCA), is of minor importance, because it only represents 3% of the total bile acid pool in humans [10]. Besides their well-established functions in resorption of lipid-soluble nutrients and cholesterol catabolism, BAs also play an important role as signaling molecules (summarized in [11]). For instance, the nuclear farnesoid X-receptor (FXR) is activated by physiological concentrations of bile salts [12]. As a nuclear receptor, it regulates multiple genes which are involved in lipid, glucose and bile acid metabolism. Notably, the activation of FXR also leads to an upregulation of apolipoprotein CII, which activates the lipoprotein lipase (LPL) [13], an enzyme that has been implicated to promote HCV entry and reduce infectivity of cell-culture derived hepatitis C virus particles (HCV_CC_) [14]. Moreover, BAs repress secretion of apolipoprotein B containing lipoproteins through inhibition of the microsomal triglyceride transfer protein (MTP) [15]. As a consequence, they may influence secretion of infectious HCV particles which depends on MTP and apoB secretion [16]. Collectively, these data suggest that endocrine functions of BAs regulate host cell pathways which may influence RNA-replication, virus production and infectivity of HCV particles and in turn treatment efficacy and viral pathogenesis.

The influence of BAs on HCV GT1 and GT2a subgenomic replicons has been reported previously [7], [17]. These data suggested that selectively GT1 was stimulated by BAs while GT2a was refractory to regulation by BAs. Moreover, it was unclear which step(s) of the viral life cycle were influenced. Therefore, we investigated the effect of BAs on different stages of the HCV replication cycle and analyzed GT-dependent viral factors essential for regulation by BAs.

## Results

### Bile acids increase HCV GT1 RNA-replication

**Figure 1:**
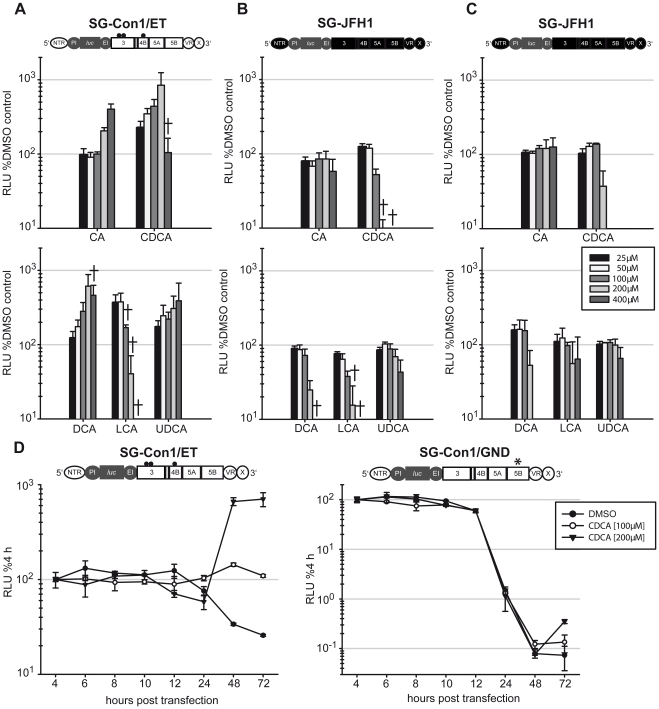
Genotype and cell type dependent influence of bile acids on HCV RNA-replication. Lunet G-luc cells [19] were transfected with either SG-Con1/ET (**A**) or SG-JFH1 replicons (**B**) and seeded on a 96-well plate. After 4 h the medium was changed and different BAs (CA = cholic acid; CDCA = chenodeoxycholic acid; DCA = deoxycholic acid; LCA = lithocholic acid; UDCA = ursodeoxycholic acid) in concentrations ranging from 25 µM–400 µM were added. 48 h later cell viability was measured by gaussia luciferase assays. The symbol † designates concentrations with a cell viability of less than 50% of the DMSO control. 72 h after electroporation cells were lysed and replication was determined using the firefly luciferase assay. Data were normalized to DMSO control. Con1-derived genome segments are depicted in white, JFH1-derived sequences in black, and non-HCV elements are depicted in grey (PI, polio IRES; EI, encephalomyocarditis virus IRES; luc, firefly luciferase). (**C**) HuH6 cells were transfected with JFH1 replicon RNA and seeded on a 96-well plate. After 4 h, BAs were added and after 72 h cells were lysed and replication was measured using the firefly-luciferase assay. **D:** Lunet G-luc cells were transfected with Con1/ET (left panel) or Con1/GND (right panel) replicons and seeded on a 12-well plate. Bile acids or DMSO were added 4 h after electroporation. Cells were lysed at given time points; luciferase activity was determined and normalized for the 4 h value. In each case mean values of triplicates and the standard deviation is given.

Regulation of HCV replication by BAs has been analyzed solely in Huh-7 cells. To exclude a cell type-dependence of the reported regulation of HCV by bile acids, we assessed the influence of BAs also using HuH6 cells, a human hepatoblastoma cell line permissive to HCV RNA-replication [18]. To control for possible cytotoxicity of BAs we employed a Huh-7-derived cell clone expressing a secreted gaussia luciferase enzyme (G-Luc) thus permitting assessment of cell density and viability which is proportional to the level of G-Luc in the culture fluid of these cells [19]. These cells were transfected with either a subgenomic Con1 replicon (SG-Con1/ET; GT1b) (Fig. 1A) or a subgenomic JFH1 replicon (SG-JFH1; GT2a) (Fig. 1B), and the influence of two different primary (top panels) or three different secondary or tertiary (bottom panels) BAs in concentrations ranging from 25 µM–400 µM was determined using firefly luciferase assays. As expected, all BAs augmented HCV GT1b RNA-replication in Lunet G-luc cells in a dose-dependent fashion. Maximal increase of replication was observed with CDCA treatment of cells reaching 9-fold higher levels compared to DMSO-treated cells at a dose of 200 µM (Fig. 1A). In HuH6 cells the enhancement of GT1b replication could not be determined by transient luciferase assays due to insufficient RNA-replication of the GT1b replicon in these cells (data not shown). In contrast, replication efficiency of the GT2a replicon was not upregulated irrespective of the BA used and of whether Lunet G-luc (Fig. 1B) or HuH6 (Fig. 1C) host cells were employed. To rule out that these differences between Con1 and JFH1 replicons was attributable to the chosen assay system (transient replication of luciferase reporter replicons) we monitored HCV RNA replication of Con1 and JFH1 selectable replicons in stable replicon cell lines in the presence or absence of 200 µM CDCA using quantitative RT-PCR (Figure S1). Comparable to the luciferase-based assay, we only observed stimulation of the Con1 replicon but not the JFH1 replicon.

To determine if RNA translation or RNA-replication of the GT1b replicon was stimulated by BAs, we transfected Lunet G-luc cells either with SG-Con1/ET or SG-Con1/GND replicons. Since SG-Con1/GND carries an inactivating mutation in the active center of the viral RNA dependent RNA polymerase NS5B, transfection of this variant was used to determine whether BAs influence viral RNA translation and/or stability independent of regulation of RNA-replication. Since BAs had no impact on luciferase expression of SG-Con1/ET or SG-Con1/GND replicons up to 24 hours after transfection, but selectively enhanced SG-Con1/ET at later time points (Fig. 1D), we conclude that BAs may stimulate RNA-replication but not translation or RNA stability of GT1b replicons.

### Influence of BAs on the complete HCV life cycle

**Figure 2:**
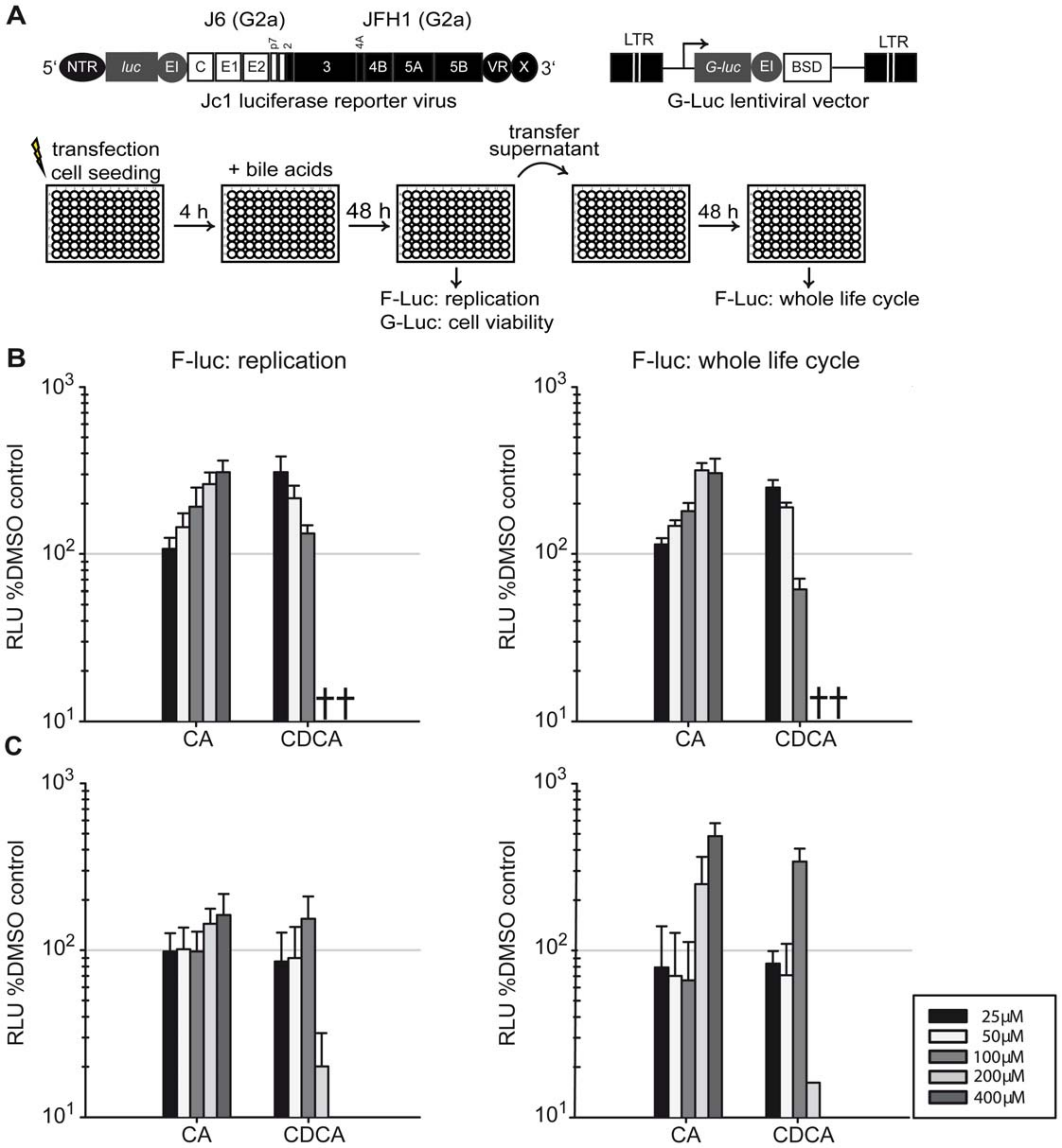
Influence of bile acids on HCV whole life cycle. **A:** Experimental setup and schematic drawing of the Luc-Jc1 reporter virus genome carrying a firefly luciferase gene and of the gaussia luciferase construct. Lunet G-luc cells were transfected with the chimeric full-length reporter virus genome and seeded on a 96-well plate. 4 h post-electroporation the medium was removed and new medium containing bile acids was added. After 48 h, RNA-replication in the transfected cells was determined by firefly luciferase assays. At the same time, culture fluid of the cells was collected to determine cell viability through G-luc activity and to inoculate naïve Lunet G-luc cells. 48 h later efficiency of virus production and infection was determined by measuring firefly luciferase in the inoculated cells. **B:** RNA-replication (left) and virus production/infection (right) in the presence or absence of given doses of BAs determined in Lunet G-luc cells. Replication and whole life cycle data were normalized to DMSO control. The symbol † designates concentrations with a cell viability of less than 50% of the DMSO control. **C:** Analysis of the influence of given BA doses on Luc-Jc1 replication in HuH6 cells (left) and on the infectivity of secreted particles upon inoculation of Lunet G-luc cells. Means values of triplicates and the standard deviation is given.

To assess the influence of BAs on the complete HCV replication cycle, we used Lunet G-luc and HuH6 cells transfected with a full-length intragenomic bicistronic full-length GT2a virus expressing firefly luciferase, designated as Luc-Jc1 [20] (Fig. 2A). Primary BAs CA and CDCA which had strongly regulated GT1b replicons were added to the cells at 4 h and their effect was determined at 48 h post transfection. At this latter time point, culture fluid of the transfected cells containing BAs and virus particles produced during the experiment was transferred to naïve cells which were analyzed 48 h later to determine possible influences of BAs on the complete virus life cycle (Fig. 2A).

CA enhanced the replication (measurement after 48 h) as well as the complete life cycle (Fig. 2B) of Luc-Jc1 up to 4-fold in a dose-dependent manner. CDCA resulted in a moderate enhancement at 25 µM and decreased replication at higher concentrations most probably due to cytotoxicity. Given that RNA-replication of the GT2a replicon was not affected by these BAs (compare Fig. 1) it was surprising that RNA-replication of the GT2a chimeric virus Luc-Jc1 was increased. To rule out that this was due to secondary rounds of infection that may be stimulated by these BAs and that may contribute to the luciferase signal determined 48 h post transfection we analyzed influences of both BAs on Luc-Jc1 transfected cells lacking CD81, an essential HCV entry factor (Lunet N cells [21]). However, even in these cells luciferase activity of Luc-Jc1 was increased by both BAs indicating that RNA-replication of the GT2a reporter virus was augmented (Figure S2). Replication was also enhanced in HuH6 cells, albeit only up to 2-fold (Fig. 2C). This finding rules out that the enhancement of Luc-Jc1 replication was cell type dependent. Since HuH6 cells cannot be infected by GT2a viruses due to low endogenous CLDN1 expression [22], we measured the infectivity of Luc-Jc1 particles produced in BA-treated HuH6 cells by inoculation of Lunet G-Luc cells and observed an up to 6-fold enhanced infectivity (Fig. 2C). Collectively, these results show that in contrast to subgenomic GT 2a constructs (Fig. 1), replication of GT 2a full length HCV was enhanced in the presence of BAs. Therefore, the enhancing effect of BAs on HCV replication is not restricted to GT1 HCV.

### Effect of BAs on HCV particle production and entry

**Figure 3:**
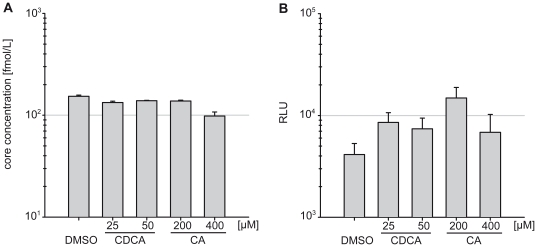
Influence of BAs on HCV particle production and infectivity. Cells were transfected, seeded and treated as described in Fig. 2. Release of core protein as a measure of viral particles in the culture fluid was determined by a commercial core-specific ELISA (**A**). Infectivity of release particles was assessed by inoculation of naïve Lunet G-luc cells (**B**). Mean values of duplicates and the standard deviation are shown.

To determine whether the enhanced HCV GT2a infectivity observed in the presence of BAs was due to the increased particle production or due to higher infectivity of the released particles, we determined extracellular levels of core protein of Luc-Jc1 transfected and BA-treated Lunet G-Luc cells. Even at higher concentrations, BAs did not elevate particle release as determined by the quantity of extracellular core protein (Fig. 3A). However, the released particles were more infectious which is evident from increased accumulation of luciferase activity in the cells inoculated with particles produced in the presence of high doses of BAs (Fig. 3B). These data indicate that BAs do not modulate the abundance of virus particles. Instead they increase infectivity either by changing particle properties, by enhancing cell entry and/or by increasing RNA-replication in the inoculated cells.

To investigate if the BAs augment virus cell entry, we used two complementary experimental systems. First, we measured the infection of retroviral HCV pseudoparticles (HCVpp) into Huh7-Lunet hCD81 G-Luc cells that had been pre-treated with CA and CDCA (Figure S3.). Since pseudoparticles consist of retroviral cores carrying HCV glycoproteins on their surface, only the early steps of virus entry are HCV dependent, i.e. virus binding, uptake and virus-membrane fusion, whereas all later steps are dependent on retroviral proteins which rigorously excludes influences of BAs on other steps of the HCV replication cycle. Notably, these particles are produced in human embryonal kidney cells (293T cells) which do not express lipoproteins and are unlikely to properly mimic possible influences of lipoproteins. Therefore, we also used single round-infectious HCV trans-complemented particles (HCV_TCP_) that are produced upon transfection of the SG-JFH1 replicon into Huh-7-derived packaging cells which express the structural proteins core, E1 and E2 as well as p7 and NS2 [23]. Since the RNA-replication of SG-JFH1 is not modulated by BAs (Fig. 1B), this experiment was aimed to reveal possible influences specifically on cell entry of HCV particles produced from lipoprotein-expressing host cells. Nevertheless, irrespective of the particle type (HCVpp or HCV_TCP_) and the duration of BA-pretreatment we did not observe a consistent and reproducible influence on HCV cell entry (Figure S3). In summary, these results indicate that BAs do not modulate production of infectious particles or cell entry of HCV in tissue cultures. Therefore, the stimulation of infection observed when GT2a particles were produced in the presence of BAs is likely due to stimulation of RNA-replication.

### Influence of viral determinants on BAs mediated stimulation of replication

**Figure 4:**
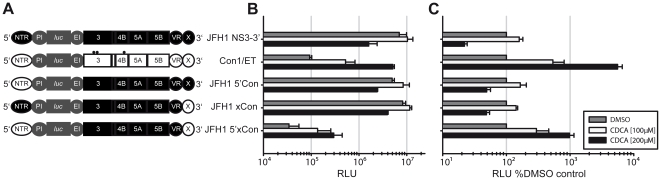
Viral determinants required for BA- mediated stimulation. **A:** Schematic representation of JFH1 NS3-3′, Con1/ET JFH1/Con1 intergenotypic chimeras. Replication enhancing mutations of Con1/ET are marked as black dots. All viral proteins were JFH1-derived and either the 5′NTR or the terminal end of the 3′ NTR (X-tail) or both were exchanged by those of Con1. **B:** Lunet G-luc cells were transfected and seeded on a 12-well plate. Indicated bile acids were added after 4 h and luciferase activity was measured 72 h post-electroporation. **C:** Data were normalized to DMSO control.

The results described above indicate that RNA-replication of GT2a replicons is not affected by BAs whereas replication of full length GT2a reporter virus genomes as well as GT1b replicons was stimulated by BAs. Notably, the latter two replicate substantially less efficiently compared with subgenomic GT2a replicons (data not shown). Therefore, it was conceivable that the highly efficient RNA-replication of the GT2a replicon masks regulation of GT2a protein function in RNA-replication by BAs. To identify viral determinants important for regulation by BAs we used intergenotypic JFH1/Con1 replicon chimeras where the 5′NTR and/or the 3′NTR X-tail sequence of JFH1 were exchanged individually or in combination with the ones of the Con1 isolate [24] (Fig. 4A). Importantly, in these constructs the protein coding sequence of the JFH1 replicon is fully conserved. As expected, the JFH1 luciferase replicon carrying Con1-derived 5′NTR and X-tail (JFH1 5′xCon) replicated less efficiently compared to the parental replicon JFH1 NS3-3′ (Fig. 4B). However, unlike the parental replicon, RNA-replication of the GT2a/1b chimeric genome was increased by addition of BAs up to 10-fold (Fig. 4B and 4C). Since insertion of the Con1 5′NTR or the Con1 X-tail alone into the JFH1 replicon did not downregulate RNA-replication efficiency and at the same time did not render these chimeras susceptible to regulation by BAs, we conclude that the Con1 genome segments individually do not confer regulation by BAs. However, GT2a replicons carrying these elements in combination are reduced in replication efficiency thus rendering these replicons responsive to BA-mediated stimulation of RNA-replication of GT2a-derived replicon proteins. Collectively, these data provide firm evidence that both GT1 and GT2a HCV are regulated by BAs.

### BAs stimulate replication of Con1 genomes with or without replication enhancing mutations

**Figure 5:**
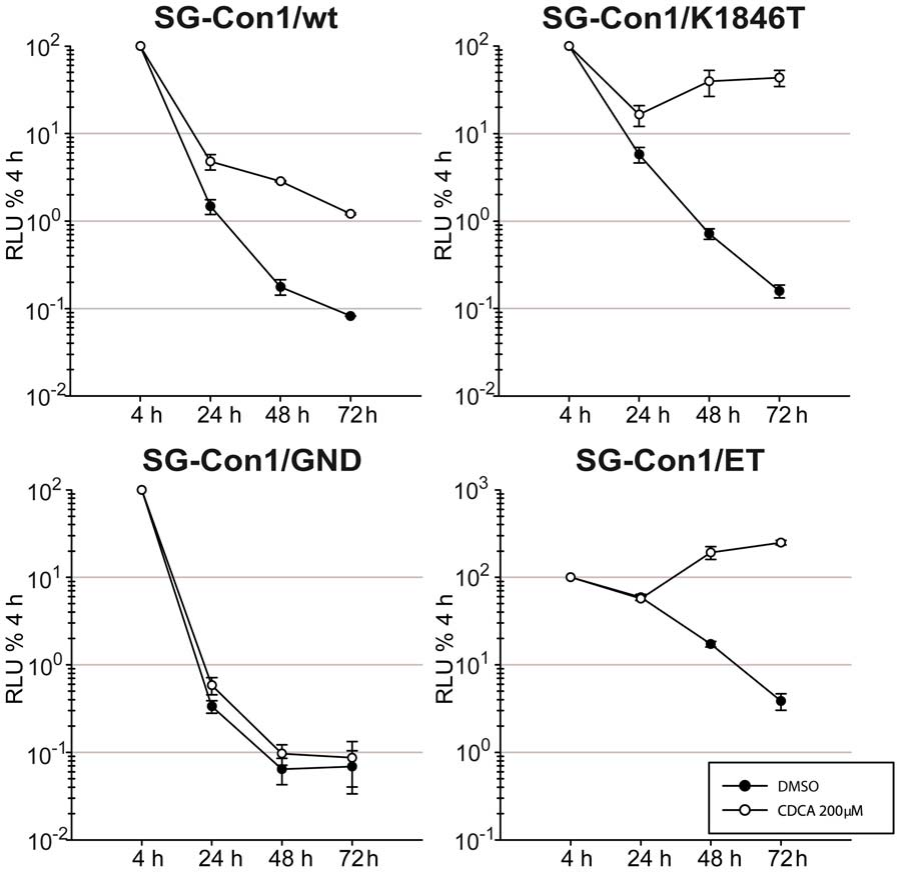
CDCA increases replication of Con1 wild type and cell culture adapted replicons. Given subgenomic Con1 replicons with or without replication enhancing mutations were transfected into Lunet G-Luc cells. At 4 h post transfection cells culture media were replaced with culture fluid with or without 200 µM CDCA. RNA replication was determined by luciferase assays and is expressed relative to the luciferase activity determined 4 h post transfection.

**Figure 6:**
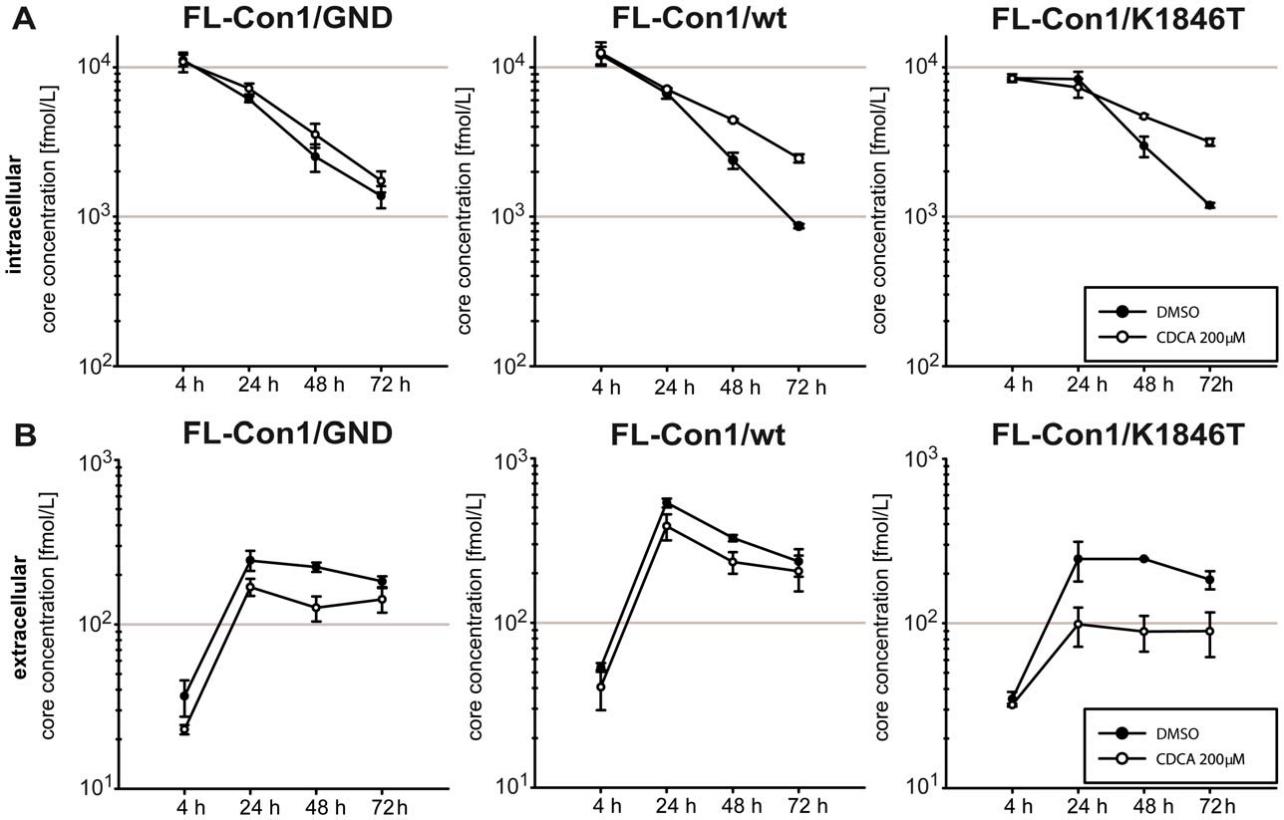
CDCA stimulates replication of full length Con1 genomes with or without adaptive mutations. Given full length Con1 genomes were transfected into Lunet G-Luc cells. At 4 h post transfection cells culture media were replaced with culture fluid with or without 200 µM CDCA. Intracellular (A) and extracellular (B) levels of HCV core protein reflecting viral translation/RNA replication and secretion of virions, respectively, were determined using a commercial ELISA.

With the exception of JFH1, most HCV consensus genomes replicate poorly in cell culture requiring specific replication enhancing mutations (REMs) to increase replication to levels sufficient for experimental analyses. Notably, at least in case of Con1, many of these REMs interfere with production of infectious progeny particles and highly cell culture adapted Con1 genomes are attenuated in vivo [25], [26]. Given these circumstances we wanted to explore if BAs can be used to stimulate replication of wild type HCV genomes that have poor replication efficiency in cell culture. To this end we transfected Lunet G-Luc cells with subgenomic Con1 luciferase replicons carrying either wild type NS3 to NS5B proteins, a single REM in NS4B (K1846T) or the highly adapted replicon with mutations in NS3 and NS4B (E1202T, T1280I, K1846T), designated ET. Interestingly, culturing cells until 72 h post transfection in the presence of 200 µM CDCA increased replication of the wild type luciferase replicon ca. 10-fold, the highly adapted ET genome ca. 50-fold and the K1846T-adapted replicon more than 200-fold (Figure 5). Next we explored if CDCA also stimulates replication of full length Con1 (FL-Con1) genomes with our without REMs and if this facilitates production of infectious viral progeny and quantification of infection events in cell culture. Thus, wild type full length Con1 (FL-Con1/wt), FL-Con1/K1846T and as reference the replication defective FL-Con1/GND mutant were transfected into Lunet G-Luc cells. Subsequently transfected cells were cultured in the presence or absence of 200 µM CDCA (Figure 6). Replication of both wild type and K1846T genomes was slightly increased by CDCA as is evident from ca. 2-fold higher levels of intracellular core protein at 72 h post transfection. Notably, the slight increase of intracellular core when cells were cultured with CDCA did not result in higher levels of secreted core protein. In contrast both for wild type and the K1846T mutant ca. 2-fold lower amounts of core protein were detectable in the culture fluid. Collectively, these data indicate that CDCA slightly stimulates replication of these full length Con1 genomes at the expense of slightly decreased release of core protein.

**Figure 7:**
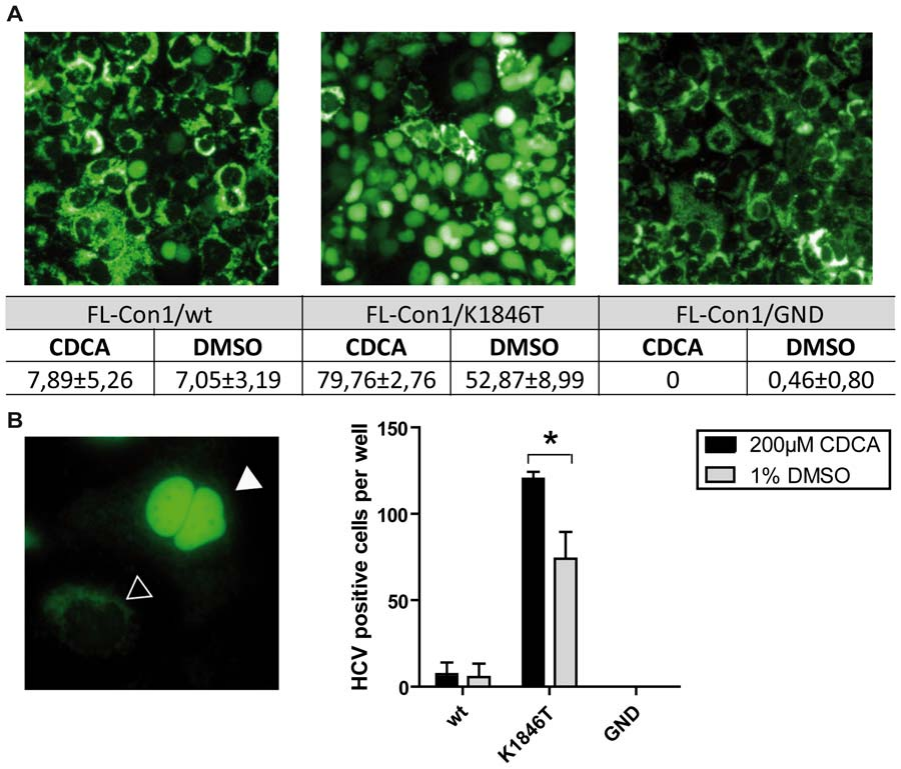
Detection of HCV replication and virus infectivity in the presence of CDCA using Lunet GFP-NLS-MAVS- reporter cells. A: Lunet MAVS-GFP reporter cells were transfected with given HCV Con1 full length genomes. Ten days after transfection relocalization of GFP to the nucleus was assessed by fluorescence microscopy. Numbers below the panels depict the percentage of cells displaying GFP in the nucleus +/− standard deviation. B: Lunet cells were transfected with given genomes, co-seeded with naïve Lunet GFP-NLS-MAVS (1∶1) and cocultured in the presence or absence of CDCA cells for four days. Subsequently the number of cells showing a nuclear localized GFP was determined by counting of 50 randomly chosen microscopic fields. The left panel shows an example of two infected cells (white arrow) displaying nuclear localized GFP and a non-infected cell (black arrow) after co-culturing with Lunet cells transfected with Con1/K1846T. The right panel depicts mean values and standard deviations from 2 independent experiments. A significant difference in infection efficiency by addition of 200 µM CDCA is indicated by an asterisk (p = 0.0486).

Similar results were obtained when we transfected these genomes into Lunet cells expressing the MAVS-GFP indicator of cellular HCV infection described by Jones et al [27] (Figure 7). More specifically, these cells express a GFP with nuclear localization signal fused to the C-terminus of MAVS, which includes a mitochondrial localization signal and the cleavage site for the HCV NS3/4A protease [27]. As a consequence, expression of the HCV NS3/4A protease results in cleavage of the MAVS-GFP protein and subsequent re-localization of GFP from the mitochondria into the nucleus, which is a simple biomarker to determine if a cell expresses the NS3/4A protease [27]. The impact of CDCA treatment on the number of HCV expressing cells was determined at 10 days after transfection, since the background levels of residual nuclear GFP, relocated after initial translation of NS3/4A from the transfected RNA was negligible at this time point. CDCA treatment increased the number of HCV expressing cells for the K1846T-adapted genome (79% versus 52% cells with nuclear localized GFP, Figure 7A). In contrast, for the wild type genome no significant stimulation was observed, possibly due to the very low replication of this genome.

To explore if CDCA facilitates infection of particles released after transfection of these genomes, we co-cultured Lunet cells transfected with these genomes with naïve Lunet MAVS-GFP cells. In this setup detection of nuclear localized GFP in the latter cells indicates productive infection by viral progeny produced from the transfected Lunet cells. Interestingly, four days after initiation of the co-culture we observed a few Lunet-MAVS-GFP cells with nuclear localized GFP when co-cultured with cells transfected the wild type or the K1846T-adapted genome (Figure 7B). At least in case of the K1846T mutant supplementation of CDCA conferred a moderate, but statistically significant (p = 0.0486), increase in the number of cells with nuclear GFP. These results indicate that particles produced in the presence of CDCA are infectious and that the moderate stimulatory effect of this BA on HCV replication likely facilitates detection of infected cells in the MAVS-GFP-based HCV infection bioassay.

## Discussion

The interplay between HCV and BAs has attracted considerable scientific attention. This was primarily due to the clinical observation that high serum levels of these compounds correlated with poor response rates to IFN-based therapies [5], [6], [8]. Previous reports have highlighted that at least replication of GT1 subgenomic replicons was increased by high doses of BAs [7], [17]. However, efforts to show a broader cross-genotype regulation of HCV replication by bile acids failed since except for GT2a no alternative and robust cell based HCV replication systems are available. As highly efficient JFH1-based GT2a replicons apparently did not respond to treatment with BAs it was unclear if the findings for GT1 replicons are more generally applicable for other viral strains. Likewise, it was unclear at what stage or stages of the viral replication cycle these molecules may influence HCV. Our findings indicate that conjugated and non-conjugated BAs as well as primary, secondary and tertiary BAs upregulate replication of HCV GT1b replicons in Huh-7 cells. Comparing the influence of BAs between replication competent and replication-inactive Con1-replicons, we show that the stimulation by BAs was not due to increased viral RNA stability or RNA translation. This implies that steps directly connected with RNA-replication like for instance establishment of membrane alterations for RNA-replication, recruitment of essential cellular co-factors or activity of essential viral factors are improved in the presence of BAs. More work is needed to find out by which mechanisms BAs facilitate HCV RNA-replication.

Using the intragenotypic chimeric infectious GT2a/2a chimera Jc1 we noted a moderate yet dose-dependent and reproducible stimulation of RNA-replication of this full length genome by BAs. Since this effect was maintained in cells that lack endogenous levels of CD81, a crucial cell entry factor for HCV, we can rule out that this effect was due to increased virus production and secondary rounds of infection. Further analyses established that BAs did not augment the number of secreted viruses or increase cell entry. Importantly, we used HCVpp and HCV_TCP_ particles to rule out that BAs modulate cell entry through interplay with lipoproteins which cannot be well studied with HCVpp. Although it has been described previously that high levels of BAs increase the activity of cellular lipoprotein lipases which in turn have been shown to decrease HCV infectivity [14] and despite of the observation that BAs downregulate secretion of ApoB containing lipoproteins [15] we did not find entry or assembly to be affected. This could be due to the host cells used by us expressing abundant lipoproteins and cell entry factors so that a subtle regulation of these factors by BAs may not be sufficient to have an impact on these steps of the HCV replication cycle. Alternatively, the cancer cell lines used by us may not reflect the complete spectrum of the regulatory functions on lipoprotein biosynthesis and secretion operating in vivo. Therefore, additional work is needed, ideally with primary human hepatocytes, to fully rule out that these steps of the HCV replication cycle are regulated by BAs.

In addition, we provide evidence that the regulation of HCV RNA-replication is likely not limited to GT1 isolates as described previously but also affects GT2a genomes. This conclusion is based on our observation that JFH1-based GT2a replicons are susceptible to regulation by BAs provided their extraordinary efficient RNA-replication is reduced by genetic manipulation of the non-translated regions. Importantly, we reduced RNA-replication of these replicons by manipulating viral non-coding regions to rule out that non-GT2a proteins may confer regulation by BAs to these chimeric genomes. Besides we show that each individual non-coding RNA segment derived from Con1 is not sufficient to confer regulation of GT2a genomes by BAs. Since both genomes carrying either the Con1 5′NTR or the X-tail replicate vigorously in transfected cells, we hypothesize that like for the parental GT2a replicon, the high replication efficiency masks the influence of BAs on these replicons. The conclusion that not only GT1 but also GT2a replication is enhanced by BAs in cell culture is also supported by the increased replication of full length GT2a genomes both in a Huh-7-derived cell line as well as in an alternative HCV-permissive human hepatoblastoma cell line (HuH6). Finally, we report that not only cell culture adapted Con1 genomes but also wild type Con1 replicons and full length genomes are stimulated by BAs. Moreover, this stimulation of replication – albeit moderate – did not increase release of core protein. In contrast, similar to replication enhancing mutations which lower or even prevent secretion of HCV core protein from Con1-transfected cells [26], incubation with BAs slightly decrease core release [26]. Nevertheless, at least for the K1846T-adapted genome addition of CDCA slightly increased the number of detectable HCV infected cells. In the future it will be interesting to find out if replication of viral genomes from different viral genotypes is also stimulated by BAs and by which mechanism and extent these compounds facilitate HCV RNA-replication. In this regard the moderate stimulatory effect of BAs on replication of wild type full length HCV genomes may facilitate the identification of novel primary isolates that are replication competent in cell culture. Considering that during bile duct obstructions serum levels of BAs ranging up to 400 µM are reached [28], [29], endocrine functions of BAs may in these patients at least transiently modulate virus replication. This in turn may influence treatment response and the ability of the virus to acquire drug resistance and should be considered when tailoring optimal treatment for patients with these complications.

## Materials and Methods

### Plasmids and replicons

The plasmids pFK-Luc-Jc1 [20], pFK-I341PI-Luc/NS3-3/JFH1, pFK-I341PI-Luc/NS3-3/Con1/ET (replicon with E1202T, I1280T and K1846T mutations), pFK-I341PI-Luc/NS3-3/Con1/E (replicon with K1846T mutation), pFK-I341PI-Luc/NS3-3/Con1/GND [18], the intergenomic genotype 1b/2a (Con1/JFH1) replicons [24], pFK-Con1/wt, pFK-Con1/K1846T and pFK-Con1/GND [26]have been described previously. pWPI-GFP/MAVS-BLR was generated by cloning a GFP gene fused to a nuclear localization signal of SV40 large T antigen and the C-terminal membrane insertion sequence of MAVS (also known as Cardif, VISA and IPS-1) into pWPI-BLR [30], a selectable derivative of the bicistronic lentiviral vector pWPI (a gift from Didier Trono). This construct was used to generate cell lines constitutively expressing a GFP-NLS-MAVS fusion protein to monitor HCV infection upon NS3/4A cleavage, as described recently [27] .

### Cell culture

Huh7-Lunet N#3 hCD81 Gaussia luciferase (designated Lunet G-luc cells here) [19], Huh7-Lunet N#3 [21], HuH6 [18] and 293T (were obtained from the ATCC; ATCC-Number: CRL-11268) [31] cells were grown in Dulbecco's modified Eagle's medium (DMEM; Invitrogen, Karlsruhe, Germany) supplemented with 2 mM L-glutamine, non-essential amino acids, 100 U of penicillin per ml, 100 µg of streptomycin per ml and 10% fetal calf serum (DMEM complete). Lunet hCD81-GFP-NLS-MAVS cells, constitutively expressing human CD81 and a GFP-NLS-MAVS fusion protein were cultured in DMEM complete supplemented with 1 mg/ml G418 and 5 µg/ml blasticidine to maintain expression of the transgenes.

### *In vitro* transcription and electroporation

*In vitro* transcription and transfection by electroporation was performed as described previously [32].

### Whole life cycle dual luciferase assay

The whole life cycle dual luciferase assay was performed as described recently [19] in the presence of different concentrations of bile acids solved in DMSO. The DMSO concentration was 1%.

### Preparation of retroviral pseudoparticles

Murine leukemia virus (MLV) -based retroviral pseudoparticles were generated as described previously [19] using pcDNA3ΔcE1E2-J6, pcz-VSV-G or pHIT456 plasmids expressing HCV E1, E2 of the J6CF (GT2a) isolate, the G-protein of vesicular stomatitis virus, or the amphotropic envelope protein of MLV, respectively.

### Preparation of HCV trans-complementated particles (HCV_TCP_)

HCV_TCP_ were generated as described previously [32].

### Detection of HCV core protein by ELISA

The virus containing supernatant was inactivated by addition of Triton X-100 at a final concentration of 1% (v/v). The amount of released core protein was determined using the commercially available core ELISA ARCHITECT HCV core AG test (Abbott, Wiesbaden, Germany).

### Quantification of HCV RNA by real time RT-PCR

Viral RNA was prepared from cells using a Nucleo Spin RNAII kit (*Macherey-Nagel*) according to the manual's instructions. 5 µL of the RNA sample was used for HCV-specific quantitative reverse transcription-PCR (qRT-PCR) analysis using a LightCycler 480 device (*Roche*). HCV-specific qRT-PCRs were conducted in duplicate measurements as published [33] utilizing a one-step RT-PCR LightCycler 480 RNA Master Hydrolysis Probes kit (*Roche*) and the following HCV-specific probe (*Molecular Biosystems*) and primers (*MWG-Biotech*): HCVMGB2 [5′-6FAM (carboxy fluoresceine)-CACGGCTAGCTGTG-MGB-3′]; XTF5 (5′-GTGGCTCCATCTTAGCCCTAGT-3′); and HCMgR2 (5′-TGCGGCTCACGGACCTTT-3′).

To normalize for equal quantities of total RNA in the samples, the GAPDH-specific mRNA was detected in parallel employing GAPDH-specific oligonucleotides (S-GAPDH, 5′-GAAGGTGAAGGTCGGAGTC-3′; A-GAPDH, 5′-GAAGATGGTGATGGG ATTTC-3′) and a GAPDH-specific probe (*TIB Molbiol*), 640-GAPDH-BBQ probe (5′-LC640-CAAgCTTCCCgTTCTCAgCCT-BBQ-3′). Reactions were performed in three stages by using the following conditions: stage 1 (RT), 3 min at 63°C; stage 2 (initial denaturation), 30 s at 95°C; stage 3 (amplification), 45 cycles of 10 s at 94°C and 20 s at 58°C. The amount of HCV RNA was calculated by comparison to serially diluted in vitro transcripts and normalized to the amount of GAPDH, which served as a housekeeping gene. HCV Core protein within cell lysates and culture fluids was quantified with a commercially available diagnostic kit (Architect Anti-HCV; *Abbott*).

### Evaluation of HCV replication and infection using the Lunet MAVS-GFP cells

To determine viral fitness under the influence of bile acids, Lunet GFP-NLS-MAVS cells were electroporated with full length Con1/wt, Con1/K1846T or Con1/GND RNA. 4 h post transfection 200 µM CDCA or 1% DMSO were added. The cells were screened for GFP translocation to the nucleus after day 10 by fluorescence microscopy in three randomly chosen fields of vision (∼300 cells per field). To address the effect of bile acids on the generation of infectious virus, Lunet cells were transfected as described above and co-seeded with naïve Lunet GFP-NLS-MAVS cells in a 1∶1 ratio. After 4 h 200 µM CDCA or 1% DMSO were supplemented. The infection rate was determined after four days by counting cells with nuclear GFP localization in 50 randomly chosen microscopic fields (10× magnification). An unpaired t-test was performed to generate the statistics. P-values<0.05 were considered statistically significant.

## Supporting Information

Figure S1 **Influence of CDCA on Con1 or JFH1 replicon cell lines.** Stable Con1 (left) or JFH1 (right) replicon cell lines were incubated with culture fluid supplemented with CDCA at a final dose of 200 µM. Cells were collected before (0 h) or after 24, 48 and 72 h of treatment. Total RNA was prepared and the abundance of HCV RNA was assessed. HCV genome equivalents per µg of total RNA are given. Mean values of duplicate measurements including the SEM are given.
(TIF)

Figure S2 **Influence of bile acids on HCV in transfected Huh7-LunetN#3 cells.** **A:** Huh7-LunetN#3 cells lacking CD81 were transfected with Luc-Jc1. A: Replication efficiency was determined as described in Fig. 2 **B:** 48 h after transfection culture fluid was collected and used to inoculate Lunet G-luc cells. Luciferase activity was determined 48 h later.
(TIF)

Figure S3 **Influence of bile acids on HCV entry.** **A–C:** HCV GT2a pseudoparticles (**A**), VSV-G pseudoparticles (**B**) MLV pseudoparticles (**C**) or HCV_TCP_ (**D**) were used to inoculate Lunet G-luc cells pre-incubated for 24 h or 48 h with indicated bile acids. After 48 h, the cells were lysed and the firefly luciferase activity was determined.
(TIF)
