# Supplementary figures and images for: Bile Acids Specifically Increase Hepatitis C Virus RNA-Replication
Source: PLoS One. 2012 Apr 25;7(4):e36029. doi: 10.1371/journal.pone.0036029 (PMC3338857; doi:10.1371/journal.pone.0036029)

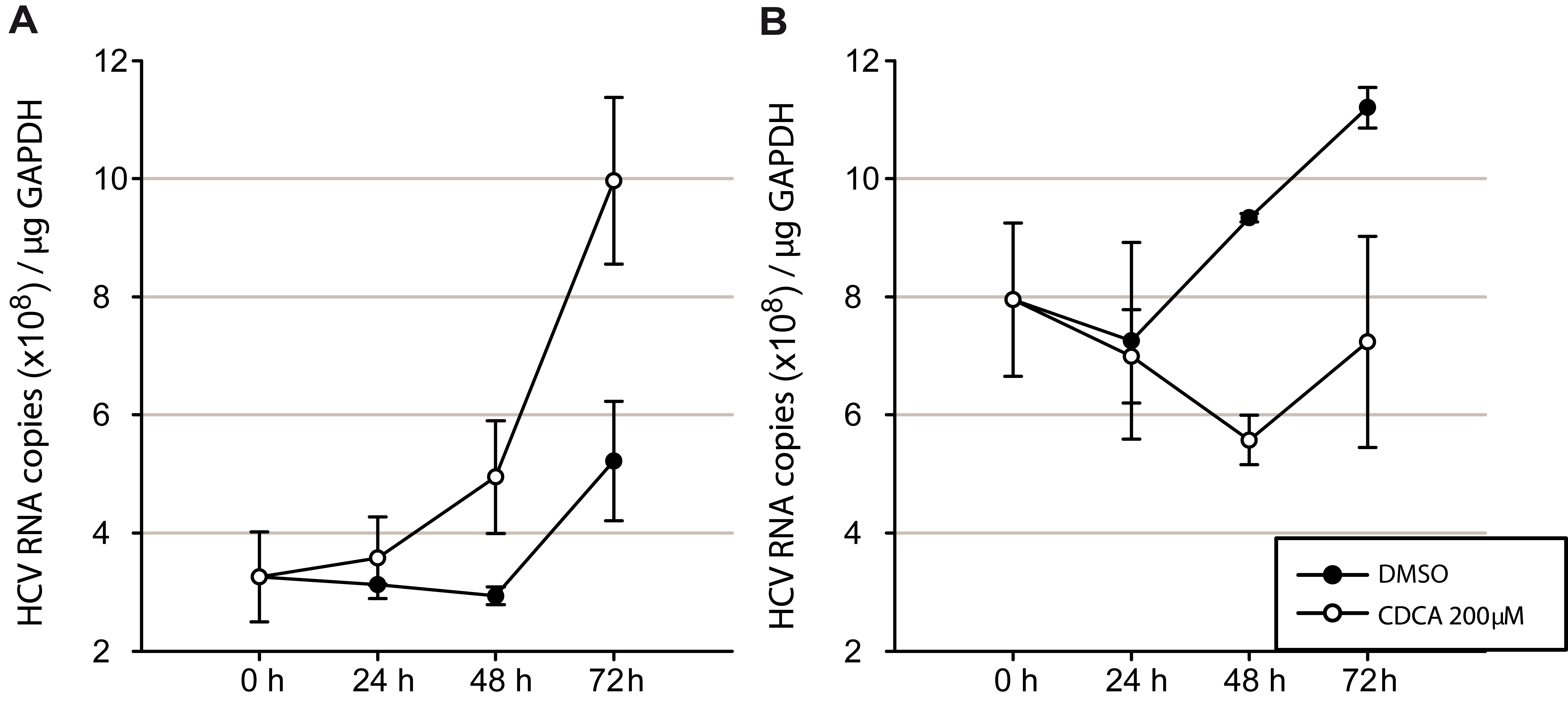

Supplement: Figure S1 — Influence of CDCA on Con1 or JFH1 replicon cell lines. Stable Con1 (left) or JFH1 (right) replicon cell lines were incubated with culture fluid supplemented with CDCA at a final dose of 200 µM. Cells were collected before (0 h) or after 24, 48 and 72 h of treatment. Total RNA was prepared and the abundance of HCV RNA was assessed. HCV genome equivalents per µg of total RNA are given. Mean values of duplicate measurements including the SEM are given. (TIF) [file pone.0036029.s001.tif]

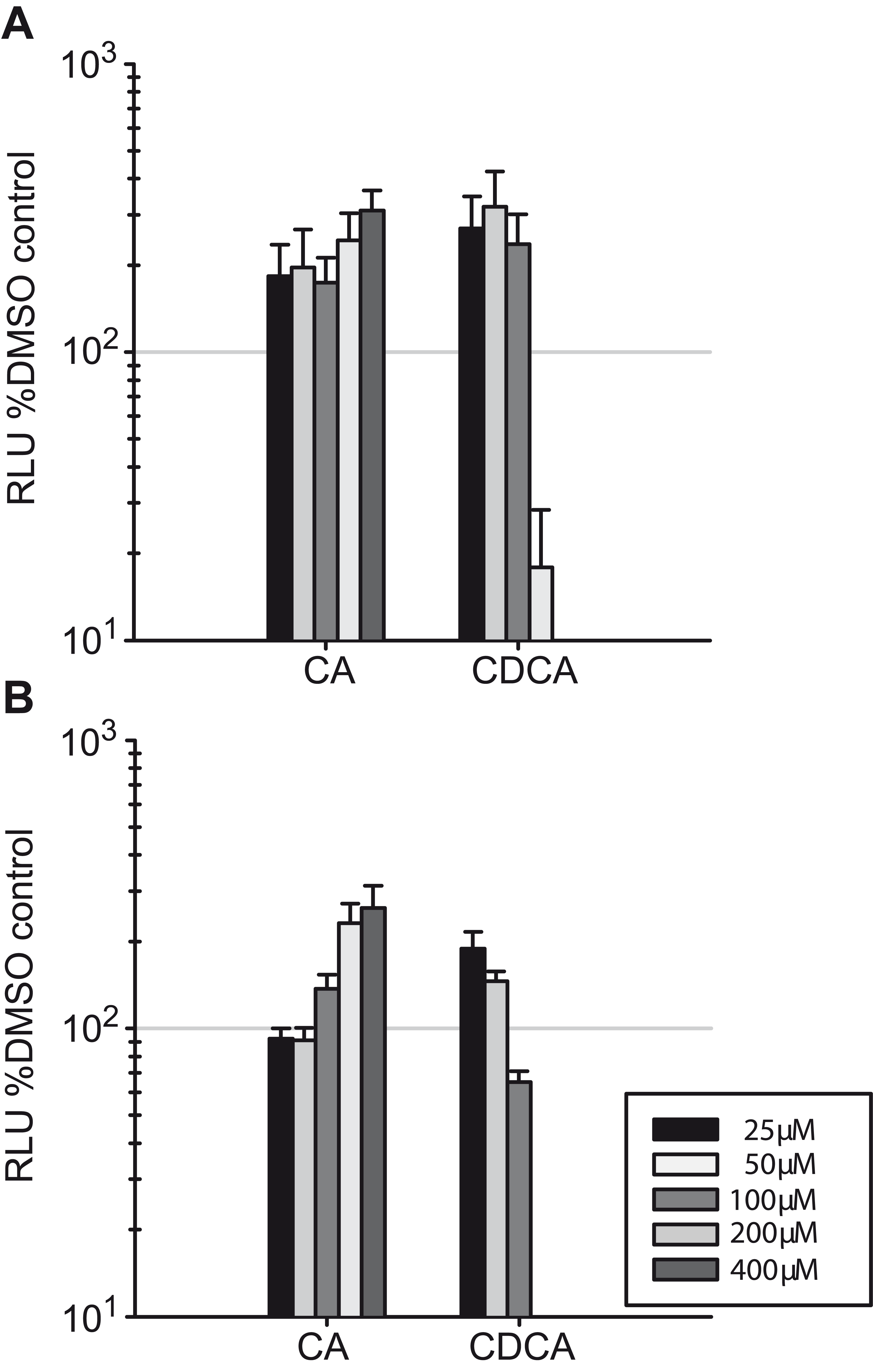

Supplement: Figure S2 — Influence of bile acids on HCV in transfected Huh7-LunetN#3 cells. A: Huh7-LunetN#3 cells lacking CD81 were transfected with Luc-Jc1. A: Replication efficiency was determined as described in Fig. 2 B: 48 h after transfection culture fluid was collected and used to inoculate Lunet G-luc cells. Luciferase activity was determined 48 h later. (TIF) [file pone.0036029.s002.tif]

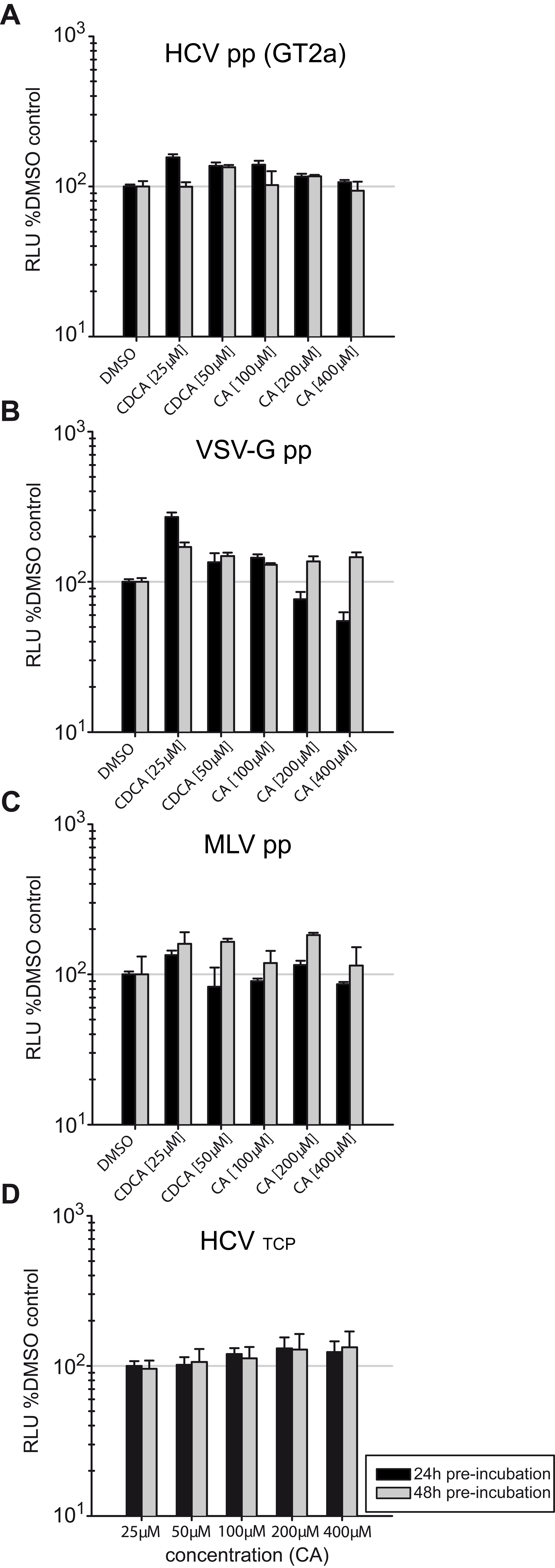

Supplement: Figure S3 — Influence of bile acids on HCV entry. A–C: HCV GT2a pseudoparticles (A), VSV-G pseudoparticles (B) MLV pseudoparticles (C) or HCVTCP (D) were used to inoculate Lunet G-luc cells pre-incubated for 24 h or 48 h with indicated bile acids. After 48 h, the cells were lysed and the firefly luciferase activity was determined. (TIF) [file pone.0036029.s003.tif]
